# Functional Response and Predation Rate of *Dicyphus cerastii* Wagner (Hemiptera: Miridae)

**DOI:** 10.3390/insects12060530

**Published:** 2021-06-07

**Authors:** Gonçalo Abraços-Duarte, Susana Ramos, Fernanda Valente, Elsa Borges da Silva, Elisabete Figueiredo

**Affiliations:** 1 Instituto Superior de Agronomia (ISA), Universidade de Lisboa, Tapada da Ajuda, 1349-017 Lisboa, Portugal; Susi_1304_@hotmail.com (S.R.); fvalente@isa.ulisboa.pt (F.V.); elsasilva@isa.ulisboa.pt (E.B.d.S.); 2 Linking Landscape, Environment, Agriculture and Food (LEAF), Instituto Superior de Agronomia, Universidade de Lisboa, Tapada da Ajuda, 1349-017 Lisboa, Portugal; 3 Forest Research Centre (CEF), Instituto Superior de Agronomia (ISA), Universidade de Lisboa, Tapada da Ajuda, 1349-017 Lisboa, Portugal

**Keywords:** biological control, dicyphini, *Bemisia tabaci*, *Ephestia kuehniella*, *Myzus persicae*, *Tuta absoluta*, protected crops, tomato

## Abstract

**Simple Summary:**

Biological control (BC) is an effective way to regulate pest populations in horticultural crops, allowing the decrease of pesticide usage. On tomato, predatory insects like plant bugs or mirids provide BC services against several insect pests. Native predators are adapted to local conditions of climate and ecology and therefore may be well suited to provide BC services. *Dicyphus cerastii* is a predatory mirid that is present in the Mediterranean region and occurs in tomato greenhouses in Portugal. However, little is known about its contribution to BC in this crop. In this study, we evaluated how prey consumption is affected by increasing prey abundance on four different prey, in laboratory conditions. We found that the predator can increase its predation rate until a maximum is reached and that prey characteristics like size and mobility can affect predation. *Dicyphus cerastii* showed high predation rates for all prey species tested, allowing us to conclude that this species is an interesting predator for BC in tomato crops.

**Abstract:**

Dicyphine mirids are important biological control agents (BCAs) in horticultural crops. *Dicyphus cerastii* Wagner can be found in protected tomato crops in Portugal, and has been observed feeding on several tomato pests. However, the predation capacity of this species is poorly studied. In order to investigate the predation capacity of *D. cerastii*, and how it is affected by prey size and mobility, we evaluated the functional response (FR) and predation rate of female predators on different densities of four prey species: *Myzus persicae* 1st instar nymphs (large mobile prey), *Bemisia tabaci* 4th instar nymphs, *Ephestia kuehniella* eggs (large immobile prey) and *Tuta absoluta* eggs (small immobile prey). Experiments were performed on tomato leaflets in Petri dish arenas for 24 h. *Dicyphus cerastii* exhibited type II FR for all prey tested. The predator effectively preyed upon all prey, consuming an average of 88.8 *B. tabaci* nymphs, 134.4 *E. kuehniella* eggs, 37.3 *M. persicae* nymphs and 172.3 *T. absoluta* eggs. Differences in the FR parameters, attack rate and handling time, suggested that prey size and mobility affected predation capacity. Considering the very high predation rates found for all prey species, *D. cerastii* proved to be an interesting candidate BCA for tomato crops.

## 1. Introduction

Tomato is an economically important crop in the Mediterranean region, in both protected and open field conditions. It is affected by several pests such as aphids (Hemiptera: Aphididae), leafminers (Diptera: Agromyzidae), mites (Acari: Tetranychidae and Eriophyidae), whiteflies (Hemiptera: Aleyrodidae), thrips (Thysanoptera), and with great importance, since its arrival in Spain in 2006, by *Tuta absoluta* (Meyrick) (Lepidoptera: Gelechiidae) [1].

The biological control of pests has been used for a long time in tomato crops. For instance, the whitefly parasitoid *Encarsia formosa* Gahan (Hymenoptera: Aphelinidae) is mass-produced and released since the 1920s [2]. More recently, tomato crops have benefited from the use of other biological control agents (BCAs) like dicyphine mirids (Heteroptera: Miridae: Bryocorinae: Dicyphini). The wide use of dicyphines is due to the fact that some of these species are zoophytophagous, which allows them to endure periods of prey scarcity by feeding on host plants, and are particularly well adapted to plants with glandular trichomes like tomato [3,4].

In the Mediterranean region, several dicyphine species in the genera *Dicyphus*, *Macrolophus*, and *Nesidiocoris* naturally occur on tomato crops [5,6,7,8], and their role against pests is widely recognized [5,9,10,11]. *Nesidiocoris tenuis* (Reuter) and *Macrolophus pygmaeus* (Rambur) are currently mass-produced and commercialized for augmentation, whereas European *Dicyphus* species provide biological control services, mostly through conservation strategies [8,12]. Despite their importance and broad use, currently commercialized dicyphines can present unfavorable aspects that limit their usefulness to biological control. Plant feeding by *Nesidiocoris tenuis*, in particular, can cause severe damage to tomato [13,14,15], while *M. pygmaeus* may take a long time to establish plentiful populations on crops [16,17]. Because of these limitations, it is important to evaluate other European dicyphines as candidate BCAs of tomato pests, as is demonstrated by the increasing research interest in species of the genus *Dicyphus* [18,19,20,21].

In Europe, the genus *Dicyphus* has 14 known species. Among them, *Dicyphus cerastii* Wagner is distributed along the Mediterranean region [21,22,23] and, in Portugal, it is commonly found in low pesticide pressure tomato greenhouses [24,25,26]. Like other dicyphines, *D. cerastii* can feed on different prey species and has been observed preying upon pests like leafminers [24], whiteflies [25,26], *T. absoluta* [25], mealybugs (Hemiptera: Pseudococcidae) and aphids [27]. However, the extent to which this predator contributes to biological control on tomato crops is not fully understood, particularly on key indicators like prey preference, predation rate, numerical and functional response.

The functional response (FR) describes how the individual predation rate changes with increasing prey availability and it is a major element when it comes to assessing the predatory efficacy of a BCA [28]. Three types of FR were proposed by Holling [29]: type I describes a linear increase of prey consumption with increasing prey density until a maximum is reached, and is mostly associated to predators like filter feeders [30]; type II expresses a negatively density-dependent relation in which the predation rate decreases with increasing prey density and is represented by a hyperbolic curve; in type III FR, a positively density-dependent is described, in which the predation rate first increases at lower prey density and then decreases at higher prey density resulting in a sigmoidal curve. Despite some records of type III FR [31,32,33], dicyphine predators are more commonly reported to have type II FR [19,32,33,34,35,36,37,38].

Type II FR is associated with unstable predator–prey dynamics [39,40], since at lower densities, there is a risk of prey extinction, as predators are able to consume most prey available. Differently, at higher densities, predators may not consume enough prey, and thus provide limited control over prey populations. This unstable dynamic leads to associating predators exhibiting type II FR to inundative biological control programs for direct pest population reduction [41], such as mirid releases in seasonal crops like tomato, rather than long-term biological control.

Functional response type and its parameters, attack rate (*a*) and handling time (*h*), are influenced by abiotic factors including spatial complexity [42] and temperature [33], and also biotic factors like the presence of alternative prey [43], prey distribution [44], and prey type and size [45].

The aim of this study was to further understand the predation capacity of *D. cerastii*, by evaluating the influence of prey size and mobility on the shape and magnitude of its FR, and on predation rate. In laboratory bioassays, *D. cerastii* females were exposed to different densities of immobile *Bemisia tabaci* (Gennadius) (Hemiptera: Aleyrodidae) nymphs and *T. absoluta* eggs, given the economic importance of these species as pests [46,47]. We also evaluated predation on, also immobile, *Ephestia kuehniella* Zeller (Lepidoptera: Pyralidae) eggs, as these are widely used as factitious prey in mirid mass rearing. And finally, predation was also evaluated for a mobile prey species, *Myzus persicae* (Sulzer) (Hemiptera: Aphididae).

## 2. Materials and Methods

### 2.1. Insects

#### 2.1.1. Predator

*Dicyphus cerastii* was originally collected from different geographical sites in Portugal. Fataca, in the south (collected from *Physalis peruviana* and *Pelargonium* sp. in gardens), Ferreira do Zêzere in central Portugal (collected on *P. peruviana* and tomato in gardens), Lisbon area (collected on tomato and *P. peruviana* in gardens), Mafra and Silveira in the Oeste region (collected on tomato greenhouses), and Póvoa de Varzim in the north (collected on tomato and tobacco in greenhouses). A mixed population colony was started from these original populations, which is frequently refreshed with wild individuals, mainly from the Oeste region. The insects used in these experiments came from this mixed population rearing kept at Instituto Superior de Agronomia (ISA). Rearing was performed in mesh cages 60 × 40 × 40 cm (Entosphinx, Pardubice, Czech Republic) set with tobacco plants about 20 cm high. To obtain young adult females (aged between 2 and 8 days after emergence) large nymphs were regularly collected from rearing cages and placed in separate cages where they were allowed to reach adulthood. The adults emerging from these cages were regularly removed and placed on a separate 35 × 35 × 35 cm cage (Entosphinx, Pardubice, Czech Republic), with access to food on tomato plants cv Montfavet (Vilmorin Iberica S.A., Alicante, Spain).

All rearing cages were kept at 25 ± 2 °C, 50 ± 20% R.H. and 14 h photoperiod, and fed a mix of eggs of *Ephestia kuehniella* Zeller (Lepidoptera: Pyralidae), and *Artemia* sp. (Anostraca: Artemiidae) cysts (Entofood^®^, Koppert Biological Systems, Berkel en Rodenrijs, The Netherlands) as factitious prey. Commercial bee pollen grains (Serramel^®^, Euromel Apicultores, Penamacor, Portugal) were also provided ad libitum (sprinkled on the leaves).

#### 2.1.2. Prey

All prey, with the exception of *E. kuehniella*, were reared at ISA’s Insectary, at room temperature (25 ± 2 °C), 50 ± 20% R.H. and a 14 h photoperiod. *Tuta absoluta* was reared from individuals collected in tomato crops from the Oeste and Alentejo regions in Portugal. Rearing units consisted of 60 × 40 × 40 cm mesh cages (Entosphinx, Pardubice, Czech Republic). In order to obtain *T. absoluta* eggs, a bouquet of fresh tomato leaves cv Montfavet (Vilmorin Iberica S.A., Alicante, Spain) was placed in plastic cups (125 mL) with water. Leaves were offered the day before bioassays to avoid egg hatching during the experimental period. Using a thin brush, fresh *T. absoluta* eggs were carefully placed on tomato leaflets to be used in experiments the same day.

*Bemisia tabaci* individuals were originally collected on *Gerbera* sp. crops in Montijo, Portugal. Colonies were kept in 60 × 40 × 40 cm mesh cages on cabbage *Brassica oleracea* L. cv Acephala. Every two weeks, new plants were placed inside rearing cages. To prevent the emergence of adults during the experimental period, only early 4th instar *B. tabaci* nymphs [48] were used. For this, after selection under a stereoscopic microscope (SMZ-2B, Nikon, Tokyo, Japan), nymphs were carefully detached and transferred onto tomato leaflets using a small brush or needle, to be used in experiments the same day.

*Myzus persicae* individuals, collected from rose plants in Lisbon, were mixed with others provided by Koppert España SL. and were reared in 60 × 40 × 40 cm mesh cages on pepper *Capsicum annuum* L. cv Piccante di Cayenna. Small nymphs (1st instar), were collected from pepper leaves with a fine brush and allowed to settle on tomato leaflets in the day of experiments.

Defrosted *E. kuehniella* eggs were obtained from the commercial product Entofood^®^ (Koppert Biological Systems, Berkel en Rodenrijs, The Netherlands) and only intact, undamaged eggs, were selected and placed on the tomato leaflets using a brush.

In order to consider prey dimensions, prey mass was obtained by weighing three groups of 50 individuals for each prey, using an AE200 scale (Mettler Toledo GmbH, Greiffensee, Switzerland), with a precision of 0.1 g. Prey body size was obtained from published literature.

### 2.2. Functional Response Bioassays

In this study, female predators were used, as predatory heteropteran females have to mate and feed in order to mature and produce eggs [49], and their predation is often higher than that of males [9,35]. Young *D. cerastii* females, between 2 and 8 days after emergence, collected from the adult cage, were individually starved in 15 mL plastic vials capped with moist cotton wool, for 24 h, at 25 ± 2 °C, to reduce differences related to varying hunger level.

Experimental arenas consisted of vertically inverted plastic Petri dishes (90 mm Ø, 15 mm high). A hole (5 mm Ø) was drilled on the top half of the dish and sealed with loose cotton wool to allow ventilation during experiments. The Petri dish was lined with one piece of filter paper (90 mm Ø) that was moistened with ca. 1 mL of water. This amount of water allowed sufficient leaf turgor during experiments. One or two (in higher prey densities) tomato leaflets cv Montfavet (Vilmorin Iberica S.A., Alicante, Spain) about 6–7 cm in length, were placed at the center of the dish, abaxial side up. During the bioassays, the arenas were sealed with Parafilm M^®^.

Each prey species was offered according to the densities in Table 1. Prey density was determined by preliminary tests performed to assess the upper predation limit for each prey, and to identify the signs of predation by mirids. Consumed prey was recognized as fully sucked prey items, when the predator left only a transparent empty chorion, in the case of eggs, or exoskeleton in the case of nymphs.

A single *D. cerastii* female was introduced into each arena and the number of consumed prey was counted after 24 h, under a stereoscopic microscope at a magnification of 50×. Consumed prey was not replaced during the experiments. The bioassays were performed in a climatic chamber (Fitoclima S600; Aralab, Rio de Mouro, Portugal) at 25 ± 1 °C, 60 ± 10% R.H., and a 14 h photoperiod.

### 2.3. Data Analysis

All FR data analyses were done in R [50] with the RStudio software [51] using the package “FRAIR” [52]. First, data were visually inspected resulting in rejection of type I functional response. In order to determine which functional response model (between type II and III) best represented the data, we fitted candidate models applying the frair_fit function that uses maximum likelihood estimation (with a binomial likelihood function) to obtain parameter estimates of the non-linear models.

For the type II functional response model, and considering that prey depletion occurred during the experiment, Rogers’ random predator equation [53] (Equation (1)) was used:

(1) $$N_{e}=N_{0}\left(1-\exp\left(a\left(N_{e}h-T\right)\right)\right)$$

where *N_e_* is the number of consumed prey, *N*_0_ is the original prey density, *a* is the attack rate, *h* is handling time and *T* is experimental period (days).

For the type III functional response model, and also considering prey depletion, we used the Hassel’s type III extension to Rogers’ random predator equation [54]. The number of consumed prey (*N_e_*) follows the same relationship defined for Rogers’ type II model, but the attack rate (*a*) is assumed to vary with prey density in the following hyperbolic function (Equation (2)):

(2) $$a=bN_{0}/\left(1+cN_{0}\right)$$

where *b* and *c* are coefficients to be fitted and *N_0_* is the original prey density.

To select the best model between type II and III FR, we followed the general approach proposed by Okuyama [55] that suggests model selection by the application of a model selection index. In our case, the fitted models were compared using the Akaike information criterion (AIC), considering that the best model has the lowest AIC, and that a ∆AIC ≤ 2 between two fits indicates that both corresponding models fit the data well [56].

In addition to the AIC approach, we used the method proposed by Juliano [57] to distinguish the overall shape of FR curves, using the frair_test function. This method consists of fitting a polynomial logistic function of the proportion of prey consumed (*N_a_/N*_0_) (Equation (3)) that, at lower prey densities, is more suitable to detect slight differences in curve shape between type II and III, than a non-linear curve [57].

(3) $$\frac{N_{e}}{N_{0}}=\frac{\exp\left(P_{0}+P_{1}N_{0}+P_{2}{N_{0}}^{2}\right)}{1+\exp\left(P_{0}+P_{1}N_{0}+P_{2}{N_{0}}^{2}\right)}$$

where *N_e_* is the number of prey consumed, *N_0_* is the initial prey density, and *P*_0_, *P*_1_, and *P*_2_ are the constant, linear, and quadratic coefficients.

The sign and significance of these coefficients determine the type of functional response: significant negative linear coefficient indicates a type II functional response (declining proportional prey consumption with increasing prey density), and significant positive linear and negative quadratic coefficients suggest a type III functional response (initial increase and subsequent decrease in proportional prey consumption) [52,57].

Finally, to compare the fitted coefficients, 95% confidence intervals (CIs) were generated by nonparametric bootstrapping using the frair_boot function; parameters with non-overlapping 95% CIs are considered significantly different [52]. Functional response curves were plotted with their respective 95% CIs using the drawpoly function.

In order to compare predation rates among the same prey density, we used the R package “FSA” [58,59] to perform a Kruskall–Wallis test, followed by Dunn’s multiple comparisons with *p*-values adjusted with the Holm method.

## 3. Results

Prey mass was directly related to its body size. *Tuta absoluta* eggs were the lightest prey (Table 2) as, on average, each egg is 0.36 mm long and 0.22 mm in diameter [60]. The remaining three prey species had similar masses (Table 2) and body sizes, as *E. kuehniella* eggs are 0.58 mm long and 0.33 mm wide [61], *Bemisia tabaci* 4th instar nymphs are approximately 0.63 mm long and 0.39 mm wide [62], and *Myzus persicae* 1st instar nymphs are 0.78 mm long and 0.33 mm wide [63].

*Dicyphus cerastii* readily accepted all prey species tested. Average consumption increased with prey availability, reaching a maximum of 88.8 *B. tabaci* 4th instar nymphs at a density of 200, 134.4 *E. kuehniella* eggs at a density of 300, 37.3 *M. persicae* 1st instar nymphs at a density of 100 and 172.3 *T. absoluta* eggs at a density of 200 (Table 3).

**Figure 1:**
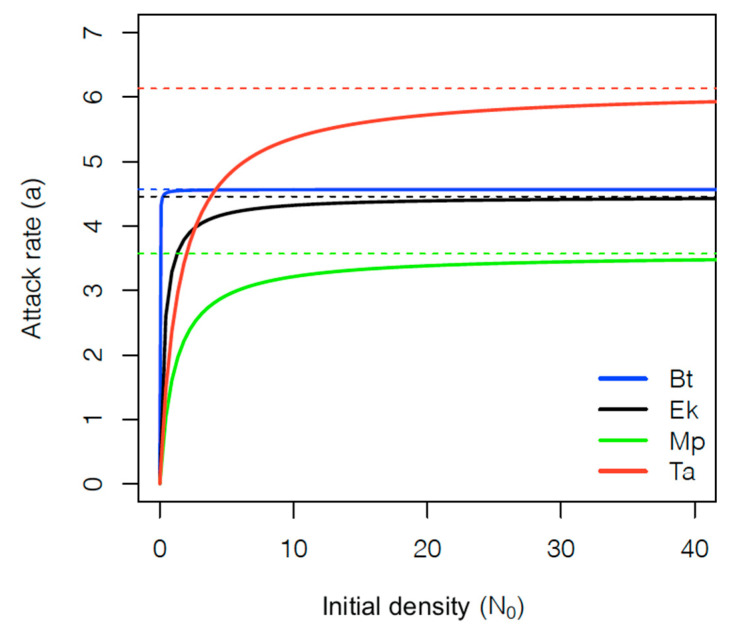
Attack rate as a function of initial prey density estimated by the Hassel’s extension for type III functional response (Equation (2)) of *Dicyphus cerastii* females preying on *Bemisia tabaci* 4th instar nymphs (Bt), *Ephestia kuehniella* eggs (Ek), *Myzus persicae* 1st instar nymphs (Mp) and *Tuta absoluta* eggs (Ta). Dotted lines represent the asymptote of the hyperbolic function (*b*/*c*) for each prey.

**Figure 2:**
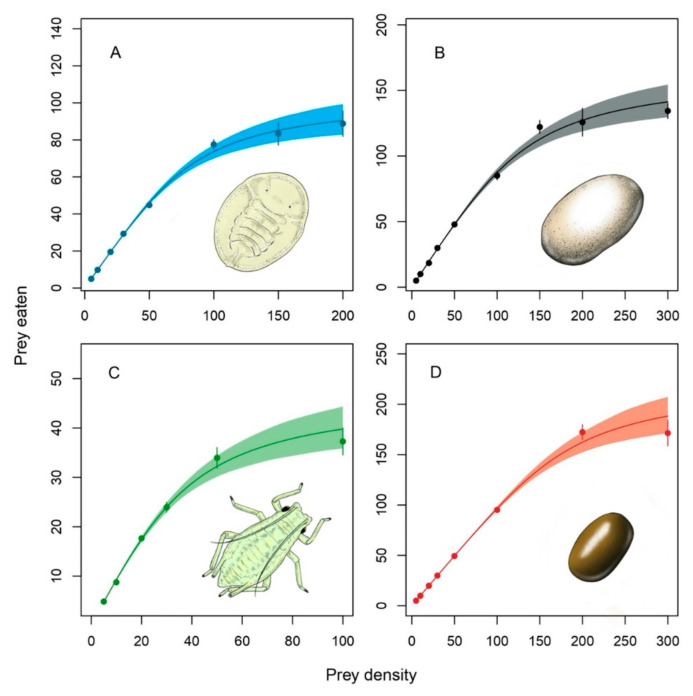
Functional response curves of *Dicyphus cerastii* females when preying on *Bemisia tabaci* 4th instar nymphs (**A**), *Ephestia kuehniella* eggs (**B**), *Myzus persicae* 1st instar nymphs (**C**) and *Tuta absoluta* eggs (**D**). Dots represent the average consumption and bars the respective standard error. Shaded areas represent bootstrapped 95% confidence intervals. Note that vertical and horizontal axis scales are not the same among prey species.

The results of fitting Rogers’ random predator equation (type II FR) and Hassel’s extension for type III are presented in Table 4 and Table 5, respectively. The type II FR model, showed lower AIC values than type III, for each of the four prey. However, ∆AIC values indicate that both models could describe the data well (Table 6). The highest ∆AIC was found for *B. tabaci*, and coherently, a plot of attack rate, as fitted by Hassel’s extension for type III FR, reveals that the attack rate of *B. tabaci* quickly tends to the asymptote of the hyperbolic function (*b*/*c*) that is very close to the value of attack rate obtained from fitting Rogers’ random predator equation (Figure 1, Table 4). In the remaining prey species (with lower ∆AIC values), this tendency is also observed, although not as fast as in *B. tabaci* (Figure 1). Despite this, the logistic regression of the proportion of prey consumed derived significant linear coefficients for all prey species (Table 7), which indicates that a type II FR should be preferable in our case, as presented in the fitted curves (Figure 2).

Estimated parameters for the Rogers’ random equation (Table 4) reveal that attack rate (*a*) was highest on *T. absoluta* eggs, followed by *B. tabaci* nymphs, *E. kuehniella* eggs and *M. persicae* nymphs. Handling time (*h*) was highest on *M. persicae* nymphs, followed by *B. tabaci* nymphs, *E. kuehniella* eggs, and *T. absoluta* eggs. From handling time, it was possible to calculate the theoretical maximum predation rate (1/*h*), which was 225.9 *T. absoluta* eggs day^−1^, 165.1 *E. kuehniella* eggs day^−1^, 104.2 *B. tabaci* nymphs day^−1^, and 47.1 *M. persicae* nymphs day^−1^. Fitting Hassel’s extension for type III also resulted in similar estimates for handling time compared to type II (Table 5).

**Figure 3:**
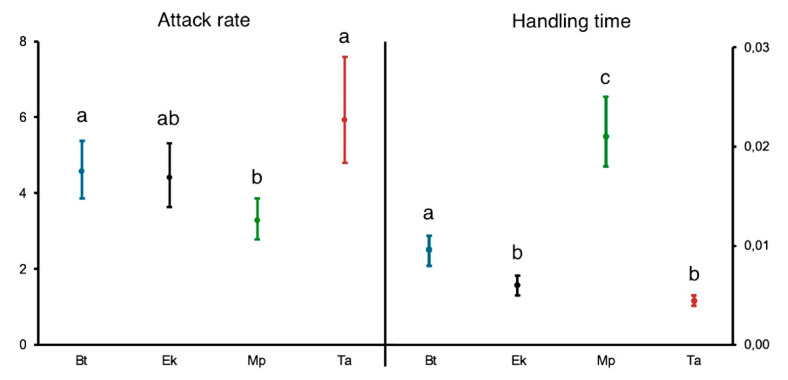
Estimates of functional response parameters attack rate, and handling time for *Dicyphus cerastii* females feeding on *Bemisia tabaci* 4th instar nymphs (Bt), *Ephestia kuehniella* eggs (Ek), *Myzus persicae* 1st instar nymphs (Mp) and *Tuta absoluta* eggs (Ta). Error bars represent bootstrapped 95% confidence intervals (CIs). In each parameter, overlapping 95% CIs are represented with the same letter. Note the different vertical axis scale.

According to the bootstrapped 95% CIs of parameter estimates (Figure 3, Table S1), attack rate did not differ between *B. tabaci* nymphs, *E. kuehniella* eggs and *T. absoluta* eggs as the correspondent 95% CIs overlapped, and the attack rate of *M. persicae* only overlapped with that of *E. kuehniella*. Handling times were different for all prey, except for *E. kuehniella* and *T. absoluta* eggs, in which 95% CIs briefly meet at 0.005 (Table S1).

## 4. Discussion

In this study, *D. cerastii* females were offered prey belonging to different species, with different characteristics of mobility and size. We found that *D. cerastii* females were able to consume the majority of prey individuals at lower densities, but predation rate decelerated as prey density increased, thus showing a type II FR for all prey tested (Table 3, Figure 2). This agrees with previous records of other dicyphine species feeding on *E. kuehniella* [34] and *T. absoluta* eggs [19,32,34], whitefly nymphs [33,35,36] and aphids [37,38,64].

The FR parameters, attack rate (*a*) and handling time (*h*), determine the slope and the height of the FR curve, respectively [65]. The attack rate is a measure of a predator’s efficiency in finding prey at low prey densities, whereas handling time is defined as the time a predator stops searching for prey after a capture [65]. In this study, prey mobility greater than prey size may have negatively affected the predator’s efficiency at lower densities, as the mobile prey, *M. persicae*, had the lowest attack rate and the values of 95% CIs for attack rate overlapped among immobile prey (*E. kuehniella*, *B. tabaci*, *T. absoluta*). However, mobility could not explain why the attack rate of *M. persicae* marginally overlapped with that of *E. kuehniella* (Figure 3, Table S1).

Prey size influences handling time, as bigger prey may require more time for manipulation and feeding [66]. Accordingly, the lowest handling time was found on the smaller prey that we offered; *T. absoluta* eggs. Despite the size difference, and similarly to attack rate, the 95% CI estimate for handling time of *T. absoluta* overlapped with that of *E. kuehniella* (Figure 3, Table S1). However, even when 95% CIs of parameter estimates overlap, parameters may still combine to produce differences in predicted consumption as a function of prey density [52], as was observed for these two prey species (Figure S1). Handling time was different among *B. tabaci*, *E. kuehniella* and *M. persicae*, despite their similarities in size and mass (Figure 3, Table S1). Other factors, besides prey size, can determine the feeding capacity of a predator [45] and, in our case, prey mobility could also explain the lower predation found on *M. persicae* nymphs. We observed that, at higher densities, aphid dispersion in the arenas at the end of experiments was also higher. Even though the majority was found on the leaflets, consumed aphids were found dispersed throughout the arena at higher densities. In this case, predators could have spent more time searching for prey compared to the other immobile prey offered. In addition to mobility, aphids can also present defense behaviors, such as exudate secretion, which can affect predation [67] and thus increase predator handling time. Despite both being immobile and size equivalent, *E. kuehniella* eggs and *B. tabaci* nymphs also had different maximum predation rates. This could be due to other prey features, that may limit predatory capacity, such as integument hardness [45], nutritional content [68] and prey digestion [69].

Type II FR is associated with unstable predator–prey dynamics [39,40]. However, in more natural setups, predators displaying type II FR may be under the pressure of stabilizing elements such as temperature [33], host plant species [70], presence of alternative prey [43], prey distribution [44], prey species [71], prey size [72], and spatial complexity [42], which may approach their predatory activity to a type III FR. In our case, although the combination of the AIC method with the logistic regression indicated that a type II FR model was preferable, the low ∆AIC between type II and III models suggests that the type III model could also fit well, particularly on *E. kuehniella*, *M. persicae* and *T. absoluta* (Table 6, Figure 1). This could mean that our setup may have hampered a more evident distinction between type II and III FR, as either due to its small size, or low spatial complexity, predators could have found prey unrealistically easily, particularly at low densities.

Although traditionally more associated to vertebrates, type III FR has been increasingly reported for arthropods including dicyphine predators such as *M. pygmaeus* and *N. tenuis* [31], *Engytatus varians* (Distant) and *Macrolophus basicornis* (Stål) females [32] feeding on *T. absoluta* eggs, and for *N. tenuis* on *B. tabaci* [33]. In more natural setups, like greenhouse crops, it is possible that the presence of stabilizing elements could drive dicyphine predators to display an FR closer to type III. One of such effects could be that, in more complex habitats, predation may be reduced at lower prey densities, since the ability to find prey can be affected by the availability of refuge [42,73]. Additionally, horticultural crops are often colonized by different arthropods that may be preyed upon by dicyphine predators. This abundance of alternative prey can also stabilize predator–prey systems if the predator is able to switch between available prey [43], which dicyphines, in general, do [74]. Furthermore, glandular trichome bearing plants, such as tomato, provide abundant entrapped arthropod cadavers, which may serve as lower effort prey on which these predators also feed [24]. Phytophagy may also stabilize predator-prey dynamics by helping dicyphines avoid bottom-up effects of prey scarcity. However, this effect may be more important on adult predators, as plant feeding alone may affect immature development in some species [75,76]. Additionally, some plant resources may reduce excessive top-down effects on prey and stabilize predator-prey dynamics, as was demonstrated for *M. pygmaeus*, which reduced its predation rate on *M. persicae* when eggplant flowers or pollen were available [77].

Functional response outcomes, although important in understanding predator–prey dynamics, can be difficult to interpret, and mostly give a theoretical contribution to the assessment of the biological control potential of a natural enemy. Predation rate, however, provides a concrete measure of the feeding capacity of a BCA and allows for direct comparison with other predators.

We observed that, for all prey, *Dicyphus cerastii* females were highly voracious and predation often started when Petri dishes were still being sealed. *Dicyphus cerastii* was able to consume an average of 172.3 *T. absoluta* eggs, and we estimated a maximum predation rate of 225.9 eggs day^−1^. These are higher values than those found for similar sized dicyphines like *D. bolivari* and *D. errans* that can feed on more than 130 *T. absoluta* eggs when exposed to 350 prey items, but have estimated maximum predation rates of 188.52 and 197.24 eggs for *D. bolivari* and *D. errans* females, respectively [19]. *Dicyphus cerastii* also showed higher predation than *M. pygmaeus* and *N. tenuis* which are reported to consume approximately 50 *T. absoluta* eggs daily [78]. Our results also indicate higher predation compared to neotropical mirid species: *Tupiocoris cucurbitaceus* (Spinola) could prey on an average of 147.45 eggs day^−1^ [79]; *Campyloneuropsis infumatus* (Carvalho), *Engytatus varians* (Distant) and *Macrolophus basicornis* (Stål) females consumed an average of 51.0, 91.1 and 100.8 *T. absoluta* eggs, respectively, although these experiments were carried on tomato seedlings [32].

Predation on aphids was also high, as *D. cerastii* females could consume up to an average of 37.3 *M. persicae* 1st instar nymphs day^−1^ whereas *D. tamaninii* and *M. pygmaeus* can feed on 22.8 and 21.7 1st instar nymphs of *M. persicae*, respectively [80]. *D. cerastii* also surpassed *T. cucurbitaceus* that preyed on 19.75 *M. persicae* nymphs [79], although these authors used mixed nymphal instars of the aphid (1st−3rd). In another study, *D. maroccanus* (*syn*. *D. bolivari* [21]) and *N. tenuis* females only preyed on approximately 15 1st instar nymphs of *M. persicae*, whereas *M. pygmaeus* fed on roughly 10 [81]; however, only 20 aphids were offered initially. Despite the previously mentioned lower predation rates, in experiments with different aphid species, *D. tamaninii* females fed on 46.2 young nymphs of *Aphis gossypii* Glover on cucumber, and 43.6 *Macrosiphum euphorbiae* (Thomas) on tomato [38], which suggests that predation rate may depend on aphid species.

*Dicyphus cerastii* females could prey upon an average of 88.8 *B. tabaci* 4th instar nymphs when 200 individuals were offered, which is a much higher predation rate than that found for most other dicyphine species. *Dicyphus tamaninii* is reported to prey on an average of 12 *B. tabaci* 4th instar nymphs day^−1^, whereas *M. pygmaeus* could consume 5 [82]. *Tupiocoris cucurbitaceus* females can prey on an average of 38.2 *B. tabaci* nymphs (3rd–4th instar) [79], and *N. tenuis* on up to 42.1 and 45.1 *B. tabaci* 4th instars day^−1^ at 25 °C and 35°C respectively [33]. Finally, in a study with the greenhouse whitefly *Trialeurodes vaporariorum* (Westwood), *D. errans* females were reported to have an estimated maximum predation rate of 114 4th instar prey [36], which is similar to what we found here for *D. cerastii* on *B. tabaci* (104.2 nymphs day^−1^).

The predation rate on *E. kuehniella* eggs is important for mass rearing dicyphine predators, as it is widely used as factitious prey. We found that *D. cerastii* females fed on an average of 134.4 *E. kuehniella* eggs which agrees with the predation rate previously reported for *D. hesperus* that can consume approximately 139 *E. kuehniella* eggs in 24h [83]. A lower predation has been reported for *N. tenuis*, which is able to consume 58 *E. kuehniella* eggs day^−1^ [84].

As mentioned above, our setup may have been too simple, as Petri dishes represent very simplified versions of what predators may encounter in nature. In the future, FR should be evaluated under more complex arenas, with multiple prey in order to assess the effect of stabilizing elements like spatial complexity and prey switching.

Besides FR and predation rate, there are other factors that may be important to address in future research regarding *D. cerastii*. Among these, the numerical response, or how the predator population changes with prey density [28], is of major importance to fully understand the potential of *D. cerastii* to regulate pests. In the case of type II predators, population size is crucial for the success of biological control, particularly at higher prey densities, when individual predation capacity may be limited. Moreover, numerical response is influenced by biological parameters that drive population dynamics such as reproductive and developmental thermal thresholds, and further information about these parameters is required for *D. cerastii*. Prey suitability also impacts predator populations, since different prey may have distinct impact on predator performance, as shown for *M. pygmaeus* [85,86] and *N. tenuis* [86] females that have lower fertility when feeding on *T. absoluta* eggs compared to those of *E. kuehniella*. Determining prey preference is also essential in the case of generalist predators like dicyphines, which may disperse their predation through prey switching behavior [74]. Therefore, an insight into predator preferences may help to understand and predict the efficacy of *D. cerastii* in multiple prey situations. Dicyphines can also present cannibalistic and intraguild predatory interactions [87,88]. Both these direct, and indirect interactions such as competitive displacement [31], may also affect the success of biological control programs with these predators. Finally, it is important to note that despite their services as BCAs, dicyphines can also damage crops through plant feeding [13,14,15], and the severity of plant damage is related to mirid species [14]. So far, the phytophagy of *D. cerastii* has been studied on tomato plantlets, on which it produced necrotic spots on leaves [25]. Therefore, a larger assessment on the impact of phytophagy of this species should also be considered in the future.

## 5. Conclusions

This work presents the first data on the functional response and predation rate of *D. cerastii* on four different prey species. *Dicyphus cerastii* exhibited type II FR for all prey tested. There were differences in the FR parameters, attack rate and handling time, suggesting that prey characteristics such as size and mobility, had an impact on predation capacity. Overall, *D. cerastii* was quite voracious, as higher predation rates were found for all prey compared to other predatory mirids, suggesting that this species may be relevant among dicyphine BCAs. Although our experiments were carried in small arenas, these results provide a valuable insight into the predatory capacity of *D. cerastii* on different prey, particularly on important tomato pests like *T. absoluta* and *B. tabaci*, encouraging further research on the BCA potential of this predator.

## Figures and Tables

**Table 1:** Number of replicates for each prey density offered to *Dicyphus cerastii* females.

Prey Species	Density								
	5	10	20	30	50	100	150	200	300
*Bemisia tabaci*	20	20	20	20	20	17	17	17	-
*Ephestia kuehniella*	20	20	20	20	21	17	15	15	15
*Myzus persicae*	20	20	20	20	20	15	-	-	-
*Tuta absoluta*	20	20	20	20	20	15	-	15	10

**Table 2:** Weight (mean ± standard error) of groups of 50 prey individuals (*Bemisia tabaci* 4th instar nymphs, *Ephestia kuehniella* eggs, *Myzus persicae* 1st instar nymphs or *Tuta absoluta* eggs).

Prey Species	Weight (mg)
*Bemisia tabaci*	1.13 ± 0.03
*Ephestia kuehniella*	1.27 ± 0.03
*Myzus persicae*	1.33 ± 0.03
*Tuta absoluta*	0.67 ± 0.03

**Table 3:** Number (mean ± standard error) of prey (*Bemisia tabaci* 4th instar nymphs, *Ephestia kuehniella* eggs, *Myzus persicae* 1st instar nymphs or *Tuta absoluta* eggs) consumed by *Dicyphus cerastii* females at each density in 24 h *.

Prey	Density								
	5	10	20	30	50	100	150	200	300
*B. tabaci*	5.0 ± 0.0a	9.8 ± 0.1a	19.5 ± 0.2a	29.3 ± 0.4a	44.8 ± 1.1a	77.5 ± 2.6a	83.4 ± 6.1a	88.8 ± 6.7a	-
*E. kuehniella*	5.0 ± 0.0a	10.0 ± 0.0a	18.5 ± 0.8a	29.9 ± 0.1a	47.9 ± 0.9ab	85.1 ± 3.1ab	122.1 ± 5.0b	125.8 ± 10.6b	134.4 ± 5.8a
*M. persicae*	4.9 ± 0.1a	8.8 ± 0.3b	17.7 ± 0.4b	24.0 ± 1.0b	34.0 ± 2.1c	37.3 ± 2.7c	-	-	-
*T. absoluta*	5.0 ± 0.0a	9.9 ± 0.1a	19.9 ± 0.1a	29.9 ± 0.1a	49.4 ± 0.4b	95.1 ± 1.8b	-	172.3 ± 7.4c	171.4 ± 12.6b

* Means followed by the same letter within columns correspond to groups among which values are not significantly different for Dunn’s multiple comparison test (Holm *p* > 0.05).

**Table 4:** Parameters *a* (attack rate) and *h* (handling time), standard error (S.E.) estimated by maximum likelihood using Rogers’ random predator equation (Type II functional response) for *Dicyphus cerastii* females feeding on different prey (*Bemisia tabaci* 4th instar nymphs, *Ephestia kuehniella* eggs, *Myzus persicae* 1st instar nymphs or *Tuta absoluta* eggs).

Prey	Parameter	Estimate	S.E.	Z *	*p*-Value
*Bemisia tabaci*	*a*	4.57	1.72 × 10^−1^	26.49	<0.001
	*h*	9.60 × 10^−3^	1.73 × 10^−4^	55.48	<0.001
*Ephestia kuehniella*	*a*	4.42	1.34 × 10^−1^	32.90	<0.001
	*h*	6.06 × 10^−3^	9.76 × 10^−5^	62.06	<0.001
*Myzus persicae*	*a*	3.28	1.77 × 10^−1^	18.57	<0.001
	*h*	2.13 × 10^−2^	7.66 × 10^−4^	27.73	<0.001
*Tuta absoluta*	*a*	5.93	2.11 × 10^−1^	28.18	<0.001
	*h*	4.43 × 10^−3^	7.71 × 10^−5^	57.40	<0.001

* z-statistics value to the test of the parameter difference from zero and the corresponding *p*-value.

**Table 5:** Parameters *b* and c, and *h* (handling time), and respective standard error (S.E.) estimated by maximum likelihood using Hassel’s extension to Rogers’s random predator equation (Type III functional response) for *Dicyphus cerastii* females feeding on different prey (*Bemisia tabaci* 4th instar nymphs, *Ephestia kuehniella* eggs, *Myzus persicae* 1st instar nymphs or *Tuta absoluta* eggs).

Prey	Parameter	Estimate	S.E.	Z *	*p*-Value
*Bemisia tabaci*	*b*	801.24	4.89 × 10^−10^	1.64 × 10^12^	<0.001
	*c*	175.45	2.23 × 10^−9^	7.85 × 10^10^	<0.001
	*h*	9.60 × 10^−3^	1.31 × 10^−4^	73.41	<0.001
*Ephestia kuehniella*	*b*	13.89	2.06 × 10^−2^	675.55	<0.001
	*c*	3.11	9.08 × 10^−2^	34.29	<0.001
	*h*	6.07 × 10^−3^	9.77 × 10^−5^	62.19	<0.001
*Myzus persicae*	*b*	3.25	3.86	8.40 × 10^−1^	4.01 × 10^−1^
	*c*	0.91	1.18	7.73 × 10^−1^	4.40 × 10^−1^
	*h*	2.17 × 10^−2^	9.65 x10^−4^	22.53	<0.001
*Tuta absoluta*	*b*	4.28	4.14 × 10^−3^	1033.38	<0.001
	*c*	6.98 × 10^−1^	2.46 × 10^−2^	28.38	<0.001
	*h*	4.46 × 10^−3^	7.68 × 10^−5^	58.13	<0.001

* z-statistics value to the test of the parameter difference from zero and the corresponding *p*-value.

**Table 6:** Akaike information criterion (AIC) for the two candidate functional response models for each prey (*Bemisia tabaci* 4th instar nymphs, *Ephestia kuehniella* eggs, *Myzus persicae* 1st instar nymphs or *Tuta absoluta* eggs).

Prey	Type II	Type III *
*Bemisia tabaci*	1252.89	1254.89 (2.00)
*Ephestia kuehniella*	1844.38	1846.29 (1.92)
*Myzus persicae*	698.53	699.77 (1.25)
*Tuta absoluta*	1164.98	1165.90 (0.92)

* Values in brackets represent ∆AIC: difference between AIC value for the current model and the lowest AIC value for each prey.

**Table 7:** Estimates and respective standard error (S.E.) of the linear coefficient of logistic regression analysis of the proportion of prey (*Bemisia tabaci* 4th instar nymphs, *Ephestia kuehniella* eggs, *Myzus persicae* 1st instar nymphs or *Tuta absoluta* eggs) consumed by *Dicyphus cerastii* females in 24 h.

Prey	Estimate	S.E.	Z *	*p*-Value
*Bemisia tabaci*	−1.69 × 10^−2^	4.49 × 10^−4^	−37.73	<0.001
*Ephestia kuehniella*	−1.12 × 10^−2^	2.45 × 10^−4^	−45.63	<0.001
*Myzus persicae*	−2.84 × 10^−2^	1.13 × 10^−3^	−25.18	<0.001
*Tuta absoluta*	−1.52 x10^−2^	4.36 × 10^−4^	−34.75	<0.001

* z-statistics value to the test of the parameter difference from zero and the corresponding *p*-value.

## Data Availability

The datasets analyzed in the present study are available from the corresponding authors on reasonable request.

## Supplementary Materials

The following are available online at https://www.mdpi.com/article/10.3390/insects12060530/s1, Table S1: Parameters *a* (attack rate) and *h* (handling time), standard error (S.E.) and respective bootstrapped 95% confidence intervals estimated by Rogers’s random predator equation (Type II functional response) for *Dicyphus cerastii* females feeding on different prey species (*Bemisia tabaci* 4th instar nymphs, *Ephestia kuehniella* eggs, *Myzus persicae* 1st instar nymphs or *Tuta absoluta* eggs); Figure S1: Functional response curves of *Dicyphus cerastii* females when preying on *Ephestia kuehniella* eggs (Ek) and *Tuta absoluta* eggs (Ta). Black dots represent the average consumption and bars the respective standard error. Shaded areas represent bootstrapped 95% confidence intervals.

## References

[1] Urbaneja A., Vercher R., Navarro Llopis V., Porcuna y Col J.L., García Marí F. (2007). La polilla del tomate, *Tuta absoluta*. Phytoma.

[2] Van Lenteren J. (1988). Biological and integrated pest control in greenhouses. Annu. Rev. Entomol..

[3] Voigt D. (2019). Foothold matters: Attachment on plant surfaces promotes the vitality of omnivorous mirid bugs *Dicyphus errans*. Arthropod. Plant. Interact..

[4] Wheeler A.G., Krimmel B.A. (2015). Mirid (Hemiptera: Heteroptera) specialists of sticky plants: Adaptations, interactions, and ecological implications. Annu. Rev. Entomol..

[5] Lykouressis Ρ., Perdikis D.C., Chalkia C.A. (2000). The effects of natural enemies on aphid populations on processing tomato in central Greece. Entomol. Hell..

[6] Alomar Ò., Goula M., Albajes R. (2002). Colonisation of tomato fields by predatory mirid bugs (Hemiptera: Heteroptera) in northern Spain. Agric. Ecosyst. Environ..

[7] Ferracini C., Ingegno B.L., Mosti M., Navone P., Tavella L., Alma A. (2012). Promising native candidates for biological control of *Tuta absoluta* in Italy. IOBC/WPRS Bull..

[8] Castañé C., Alomar O., Goula M., Gabarra R. (2004). Colonization of tomato greenhouses by the predatory mirid bugs *Macrolophus caliginosus* and *Dicyphus tamaninii*. Biol. Control.

[9] Ingegno B.L., Ferracini C., Gallinotti D., Alma A., Tavella L. (2013). Evaluation of the effectiveness of *Dicyphus errans* (Wolff) as predator of *Tuta absoluta* (Meyrick). Biol. Control.

[10] Calvo J., Bolckmans K., Stansly P.A., Urbaneja A. (2009). Predation by *Nesidiocoris tenuis* on *Bemisia tabaci* and injury to tomato. BioControl.

[11] Lykouressis D.P., Perdikis D.C., Konstantinou A.D. (2009). Predation rates of *Macrolophus pygmaeus* (Hemiptera: Miridae) on different densities of eggs and nymphal instars of the greenhouse whitefly *Trialeurodes vaporariorum* (Homoptera: Aleyrodidae). Entomol. Gen..

[12] Ingegno B.L., Candian V., Psomadelis I., Bodino N., Tavella L. (2017). The potential of host plants for biological control of *Tuta absoluta* by the predator *Dicyphus errans*. Bull. Entomol. Res..

[13] Arnó J., Castañé C., Riudavets J., Gabarra R. (2010). Risk of damage to tomato crops by the generalist zoophytophagous predator *Nesidiocoris tenuis* (Reuter) (Hemiptera: Miridae). Bull. Entomol. Res..

[14] Castañé C., Arnó J., Gabarra R., Alomar O. (2011). Plant damage to vegetable crops by zoophytophagous mirid predators. Biol. Control.

[15] Pérez-Hedo M., Urbaneja A., Horowitz A., Ishaaya I. (2016). The zoophytophagous predator *Nesidiocoris tenuis*: A successful but controversial biocontrol agent in tomato crops. Advances in Insect Control and Resistance Management.

[16] De Backer L., Megido R.C., Haubruge É., Verheggen F.J. (2014). *Macrolophus pygmaeus* (Rambur) as an efficient predator of the tomato leafminer *Tuta absoluta* (Meyrick) in Europe. A review. Biotechnol. Agron. Soc. Environ..

[17] Sanchez J.A., López-Gallego E., Pérez-Marcos M., Perera-Fernández L. (2021). The effect of banker plants and pre-plant release on the establishment and pest control of *Macrolophus pygmaeus* in tomato greenhouses. J. Pest Sci..

[18] Madeira F., Sossai S., Edo E., Pagès P., Levi N., Albajes R. (2019). Attractiveness of uninfested vegetables to the omnivorous predators *Dicyphus bolivari* and *D. errans* (Hemiptera: Miridae) and their relative suitability for oviposition. Biol. Control.

[19] Ingegno B.L., Messelink G.J., Bodino N., Iliadou A., Driss L., Woelke J.B., Leman A., Tavella L. (2019). Functional response of the mirid predators *Dicyphus bolivari* and *Dicyphus errans* and their efficacy as biological control agents of Tuta absoluta on tomato. J. Pest Sci..

[20] Bouagga S., Urbaneja A., Pérez-Hedo M. (2018). Comparative biocontrol potential of three predatory mirids when preying on sweet pepper key pests. Biol. Control.

[21] Sanchez J.A., Cassis G. (2018). Towards solving the taxonomic impasse of the biocontrol plant bug subgenus *Dicyphus* (*Dicyphus*) (Insecta: Heteroptera: Miridae) using molecular, morphometric and morphological partitions. Zool. J. Linnean Soc..

[22] Kerzhner I.M., Josifov M., Aukema B., Rieger C. (1999). Cimicomorpha II Miridae. Catalogue of the Heteroptera of the Palaearctic Region, Vol 3.

[23] Aukema B., Rieger C., Rabitsch W. (2013). Catalogue of the Heteroptera of the Palaearctic Region. Volume 6. Supplement.

[24] Carvalho P., Mexia A. (2000). First approach on the potential role of *Dicyphus cerastii* Wagner (Hemiptera: Miridae), as natural control agent in Portuguese greenhouses. Bull. OILB/SROP.

[25] Figueiredo E., Martins J., Matos T., Duarte G., Silva E.B., Mexia A. (2016). Mirid complex in Oeste region greenhouse—*Dicyphus umbertae* a promising biological control agent?. IOBC/WPRS Bull..

[26] Figueiredo E., Prieto R., Mexia A., Rodrigues S., Costa C.A., Godinho M.C. (2012). Mirid bugs as biological control agents in protected tomato crops in the oeste region. Acta Hortic..

[27] Francisco L. (2019). O predador *Dicyphus cerastii* (Hemiptera: Miridae): Estudo de Comportamento e Preferências Alimentares. Master’s Thesis.

[28] Solomon M.E. (1949). The natural control of animal populations. J. Anim. Ecol..

[29] Holling C.S. (1959). The components of predation as revealed by a study of small-mammal predation of the European pine sawfly. Can. Entomol..

[30] Jeschke J.M., Kopp M., Tollrian R. (2004). Consumer-food systems: Why type I functional responses are exclusive to filter feeders. Biol. Rev. Camb. Philos. Soc..

[31] Michaelides G., Sfenthourakis S., Pitsillou M., Seraphides N. (2017). Functional response and multiple predator effects of two generalist predators preying on *Tuta absoluta* eggs. Pest Manag. Sci..

[32] Van Lenteren J.C., Hemerik L., Lins J.C., Bueno V.H.P. (2016). Functional responses of three neotropical mirid predators to eggs of *Tuta absoluta* on tomato. Insects.

[33] Madbouni M.A.Z., Samih M.A., Namvar P., Biondi A. (2017). Temperature-dependent functional response of *Nesidiocoris tenuis* (Hemiptera: Miridae) to different densities of pupae of cotton whitefly, *Bemisia tabaci* (Hemiptera: Aleyrodidae). Eur. J. Entomol..

[34] Sharifian I., Sabahi Q., Khoshabi J. (2015). Functional response of *Macrolophus pygmaeus* (Rambur) and *Nesidiocoris tenuis* (Reuter) feeding on two different prey species. Arch. Phytopathol. Plant Prot..

[35] Hassanpour M., Bagheri M., Golizadeh A., Farrokhi S. (2016). Functional response of *Nesidiocoris tenuis* (Hemiptera: Miridae) to *Trialeurodes vaporariorum* (Hemiptera: Aleyrodidae): Effect of different host plants. Biocontrol Sci. Technol..

[36] Ingegno B.L., Bodino N., Leman A., Messelink G.J., Tavella L. (2017). Predatory efficacy of *Dicyphus errans* on different prey. Acta Hortic..

[37] Maselou D., Perdikis D., Fantinou A. (2015). Effect of hunger level on prey consumption and functional response of the predator *Macrolophus pygmaeus*. Bull. Insectology.

[38] Alvarado P., Baltà O., Alomar O. (1997). Efficiency of four heteroptera as predators of *Aphis gossypii* and *Macrosiphum euphorbiae* (Hom.: Aphididae). Entomophaga.

[39] Van Lenteren J.C., Bakker K. (1975). Functional responses in invertebrates. Netherlands J. Zool..

[40] Murdoch W.W., Oaten A., MacFadyen A. (1975). Predation and population stability. Advances in Ecological Research.

[41] Van Lenteren J.C. (2012). The state of commercial augmentative biological control: Plenty of natural enemies, but a frustrating lack of uptake. BioControl.

[42] Alexander M.E., Dick J.T.A., O’Connor N.E., Haddaway N.R., Farnsworth K.D. (2012). Functional responses of the intertidal amphipod *Echinogammarus marinus*: Effects of prey supply, model selection and habitat complexity. Mar. Ecol. Prog. Ser..

[43] Murdoch W.W. (1969). Switching in General Predators: Experiments on predator specificity and stability of prey populations. Ecol. Monogr..

[44] Feng Y., Zhou Z.X., An M.R., Yu X.L., Liu T.X. (2018). The effects of prey distribution and digestion on functional response of *Harmonia axyridis* (Coleoptera: Coccinellidae). Biol. Control.

[45] Kalinoski R.M., DeLong J.P. (2016). Beyond body mass: How prey traits improve predictions of functional response parameters. Oecologia.

[46] Orfanidou C.G., Pappi P.G., Efthimiou K.E., Katis N.I., Maliogka V.I. (2016). Transmission of tomato chlorosis virus (ToCV) by *Bemisia tabaci* biotype Q and evaluation of four weed species as viral sources. Plant Dis..

[47] Desneux N., Wajnberg E., Wyckhuys K.A.G., Burgio G., Arpaia S., Narváez-Vasquez C.A., González-Cabrera J., Ruescas D.C., Tabone E., Frandon J. (2010). Biological invasion of European tomato crops by *Tuta absoluta*: Ecology, geographic expansion and prospects for biological control. J. Pest Sci..

[48] Naranjo S.E., Ellsworth P.C. (2017). Methodology for developing life tables for sessile insects in the field using the whitefly, *Bemisia tabaci*, in cotton as a model system. J. Vis. Exp..

[49] Legaspi J.C., Legaspi B.C. (2008). Ovigeny in selected generalist predators. Florida Entomol..

[50] R Core Team (2020). R: A Language and Environment for Statistical Computing.

[51] RStudio Team (2020). RStudio: Integrated Development Environment for R.

[52] Pritchard D.W., Paterson R.A., Bovy H.C., Barrios-O’Neill D. (2017). FRAIR: An R package for fitting and comparing consumer functional responses. Methods Ecol. Evol..

[53] Rogers D. (1972). Random Search and Insect Population Models. J. Anim. Ecol..

[54] Hassell M.P., Lawton J.H., Beddington J.R. (1977). Sigmoid functional responses by invertebrate predators and parasitoids. J. Anim. Ecol..

[55] Okuyama T. (2013). On selection of functional response models: Holling’s models and more. BioControl.

[56] Burnham K.P., Anderson D.R. (2004). Multimodel inference: Understanding AIC and BIC in model selection. Sociol. Methods Res..

[57] Juliano S.A., Scheiner S., Gurevitch J. (2001). Nonlinear curve fitting: Predation and functional response curves. Design and Analysis of Ecological Experiments.

[58] Dinno A. (2017). dunn.Test: Dunn’s Test of Multiple Comparisons Using Rank Sums.

[59] Ogle D.H., Wheeler P., Dinno A. (2021). FSA: Fisheries Stock Analysis.

[60] (2005). Data sheets on quarantine pests, *Tuta absoluta*. OEPP/EPPO Bull..

[61] Brindley T.A. (1930). The growth and development of *Ephestia kuehniella* Zeller (Lepidoptera) and *Tribolium confusum* Duval (Coleoptera) under controlled conditions of temperature and relative humidity. Ann. Entomol. Soc. Am..

[62] Thompson W.M.O. (2000). Development, morphometrics and other biological characteristics of the whitefly *Bemisia tabaci* (Gennadius) on cassava. Insect Sci. its Appl..

[63] Sylvester E.S. (1954). Insectary life history and apterous instar morphology of *Myzus Persicae* (Sulzer) (Homoptera, Aphidae). Ann. Entomol. Soc. Am..

[64] Fantinou A.A., Perdikis D.C., Maselou D.A., Lambropoulos P.D. (2008). Prey killing without consumption: Does *Macrolophus pygmaeus* show adaptive foraging behaviour?. Biol. Control.

[65] Holling C.S. (1959). Some characteristics of simple types of predation and parasitism. Can. Entomol..

[66] Milonas P.G., Kontodimas D.C., Martinou A.F. (2011). A predator’s functional response: Influence of prey species and size. Biol. Control.

[67] Butler C.D., O’Neil R.J. (2006). Defensive response of soybean aphid (Hemiptera: Aphididae) to predation by insidious flower bug (Hemiptera: Anthocoridae). Ann. Entomol. Soc. Am..

[68] Schmidt J.M., Sebastian P., Wilder S.M., Rypstra A.L. (2012). The nutritional content of prey affects the foraging of a generalist arthropod predator. PLoS ONE.

[69] Papanikolaou N.E., Milonas P.G., Demiris N., Papachristos D.P., Matsinos Y.G. (2014). Digestion limits the functional response of an aphidophagous coccinellid (Coleoptera: Coccinellidae). Ann. Entomol. Soc. Am..

[70] Messina F.J., Hanks J.B. (1998). Host Plant alters the shape of the functional response of an aphid predator (Coleoptera: Coccinellidae). Environ. Entomol..

[71] Sarmento R.A., Pallini A., Venzon M., De Souza O.F.F., Molina-Rugama A.J., De Oliveira C.L. (2007). Functional response of the predator *Eriopis connexa* (Coleoptera: Coccinellidae) to different prey types. Brazilian Arch. Biol. Technol..

[72] Hassanzadeh-Avval M., Sadeghi-Namaghi H., Fekrat L. (2019). Factors influencing functional response, handling time and searching efficiency of *Anthocoris minki* Dohrn (Hem.: Anthocoridae) as predator of *Psyllopsis repens* Loginova (Hem.: Psyllidae). Phytoparasitica.

[73] Barrios-O’Neill D., Dick J.T.A., Emmerson M.C., Ricciardi A., Macisaac H.J. (2015). Predator-free space, functional responses and biological invasions. Funct. Ecol..

[74] Enkegaard A., Brødsgaard H.F., Hansen D.L. (2001). *Macrolophus caliginosus*: Functional response to whiteflies and preference and switching capacity between whiteflies and spider mites. Entomol. Exp. Appl..

[75] Perdikis D., Arvaniti K. (2016). Nymphal development on plant vs. leaf with and without prey for two omnivorous predators: *Nesidiocoris tenuis* (Reuter, 1895) (Hemiptera: Miridae) and *Dicyphus errans* (Wolff, 1804) (Hemiptera: Miridae). Entomol. Gen..

[76] Ingegno B.L., Pansa M.G., Tavella L. (2011). Plant preference in the zoophytophagous generalist predator *Macrolophus pygmaeus* (Heteroptera: Miridae). Biol. Control.

[77] Maselou D.A., Perdikis D.C., Sabelis M.W., Fantinou A.A. (2014). Use of plant resources by an omnivorous predator and the consequences for effective predation. Biol. Control.

[78] Urbaneja A., Montón H., Mollá O. (2009). Suitability of the tomato borer *Tuta absoluta* as prey for *Macrolophus pygmaeus* and *Nesidiocoris tenuis*. J. Appl. Entomol..

[79] López S.N., Muñoz A.O., Andorno A.V., Cuello E.M., Cagnotti C.L. (2019). Predatory capacity of *Tupiocoris cucurbitaceus* ( Hemiptera Miridae ) on several pests of tomato. Bull. Insectology.

[80] Messelink G.J., Bloemhard C.M.J., Hoogerbrugge H., van Schelt J., Ingegno B.L., Tavella L. (2015). Evaluation of mirid predatory bugs and release strategy for aphid control in sweet pepper. J. Appl. Entomol..

[81] Pérez-Hedo M., Urbaneja A. (2015). Prospects for predatory mirid bugs as biocontrol agents of aphids in sweet peppers. J. Pest Sci..

[82] Barnadas I., Gabarra R., Albajes R. (1998). Predatory capacity of two mirid bugs preying on *Bemisia tabaci*. Entomol. Exp. Appl..

[83] VanLaerhoven S.L., Gillespie D.R., Roitberg B.D. (2003). Diel activity pattern and predation rate of the generalist predator *Dicyphus hesperus*. Entomol. Exp. Appl..

[84] Malkeshi S.H., Talaei-Hassanloui R., Mohaghegh J., Allahyari H. (2017). Predation rate and prey preference of *Nesidiocoris tenuis* on *Ephestia kuehniella* and *Tuta absoluta* eggs in laboratory. BioControl Plant Prot..

[85] Sylla S., Brévault T., Diarra K., Bearez P., Desneux N. (2016). Life-history traits of *Macrolophus pygmaeus* with different prey foods. PLoS ONE.

[86] Mollá O., Biondi A., Alonso-Valiente M., Urbaneja A. (2014). A comparative life history study of two mirid bugs preying on *Tuta absoluta* and *Ephestia kuehniella* eggs on tomato crops: Implications for biological control. BioControl.

[87] Abraços Duarte G., Caldas F., Pechirra A., Borges da Silva E., Figueiredo E. (2021). Intraguild predation and cannibalism among Dicyphini: *Dicyphus cerastii* vs. two commercialized species. Entomol. Exp. Appl..

[88] Arvaniti K., Fantinou A., Perdikis D. (2019). Cannibalism among same-aged nymphs of the omnivorous predator *Dicyphus errans* (Hemiptera: Miridae) is affected by food availability and nymphal density. Eur. J. Entomol..

