# Supplementary material for: Functional Response and Predation Rate of Dicyphus cerastii Wagner (Hemiptera: Miridae)
Source: Insects. 2021 Jun 7;12(6):530. doi: 10.3390/insects12060530 (PMC8229145; doi:10.3390/insects12060530)
Supplement: Supplementary file 1 [file insects-12-00530-s001.zip › Figure S1.pdf]

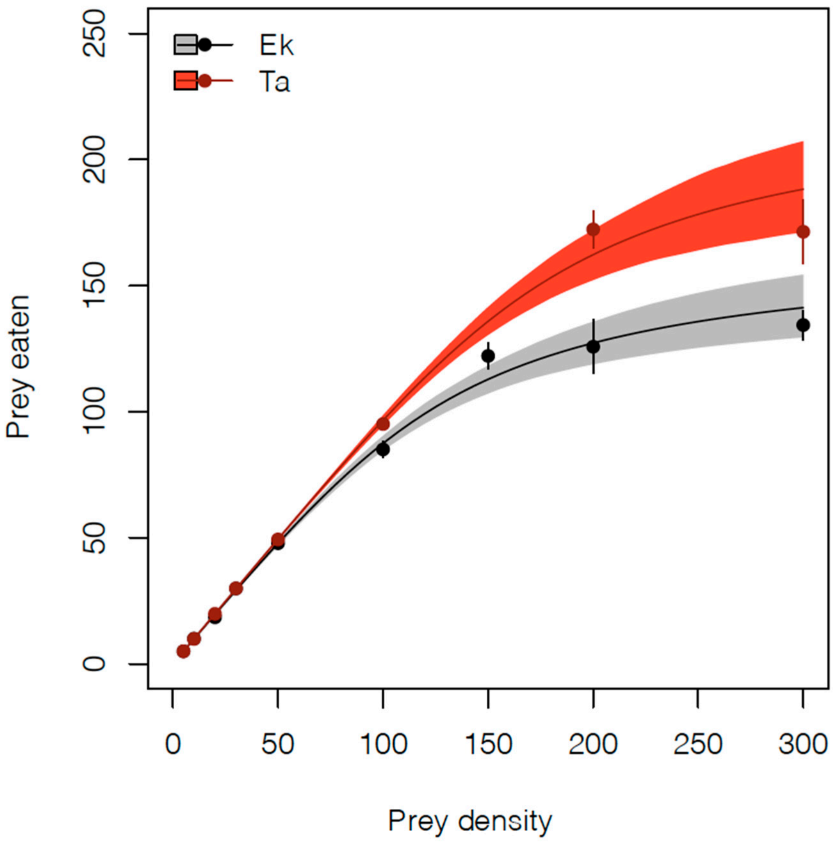

Figure S1. Functional response curves of *Dicyphus cerastii* females when preying on *Ephestia kuehniella* (Ek) and *Tuta absoluta* eggs (Ta). Dots represent the average consumption and bars the respective standard error. Shaded areas represent bootstrapped 95% confidence intervals.
