# Supplementary material for: Functional Response and Predation Rate of Dicyphus cerastii Wagner (Hemiptera: Miridae)
Source: Insects. 2021 Jun 7;12(6):530. doi: 10.3390/insects12060530 (PMC8229145; doi:10.3390/insects12060530)
Supplement: Supplementary file 1 [file insects-12-00530-s001.zip › Table S1.pdf]

Table S1. Parameters *a* (attack rate) and *h* (handling time), standard error (S.E.) and respective bootstrapped 95% confidence intervals estimated by Rogers's random predator equation (Type II functional response) for *Dicyphus cerastii* females feeding on different prey species (*Bemisia tabaci* 4th instar nymphs, *Ephestia kuehniella* eggs, *Myzus persicae* 1st instar nymphs or *Tuta absoluta* eggs).

Prey	Parameter	Estimate	S.E.	95%CI Lower	95%CI Upper	z *	*p*-Value
Bemisia tabaci	*a*	4.566	1.724×10^{-1}	3.864	5.372	26.486	<0.001
	h	9.596×10^{-3}	1.730×10^{-4}	0.008	0.011	55.481	<0.001
Ephestia kuehniella	*a*	4.416	1.342×10^{-1}	3.632	5.307	32.900	<0.001
	h	6.057×10^{-3}	9.761×10^{-5}	0.005	0.007	62.058	<0.001
Myzus persicae	*a*	3.280	1.767×10^{-1}	2.781	3.863	18.567	<0.001
	h	2.125×10^{-2}	7.662×10^{-4}	0.018	0.025	27.732	<0.001
Tuta absoluta	*a*	5.931	2.105×10^{-1}	4.789	7.597	28.179	<0.001
	h	4.427×10^{-3}	7.711×10^{-5}	0.004	0.005	57.401	<0.001

* z-statistics value to the test of the parameter difference from zero and the corresponding *p*-value
